# Investigating the intention to receive the COVID-19 vaccination in Macao: implications for vaccination strategies

**DOI:** 10.1186/s12879-022-07191-y

**Published:** 2022-03-04

**Authors:** Carolina Oi Lam Ung, Yuanjia Hu, Hao Hu, Ying Bian

**Affiliations:** 1 State Key Laboratory of Quality Research in Chinese Medicine, Institute of Chinese Medical Sciences, University of Macau, Room 2058, N22 Research Building, Macao SAR, China; 2 Department of Public Health and Medicinal Administration, Faculty of Health Sciences, University of Macau, Room 1046, E12 Research Building, Macao SAR, China

**Keywords:** COVID-19, Vaccination, Intention, Strategy, Social responsibility

## Abstract

**Background:**

Understanding the intention of receiving COVID-19 vaccines is important to inform effective vaccination strategies. This study aimed to investigate such intention, identify the key influencing factors, and determine the most important intention predictors using a theoretically principled model.

**Methods:**

An online, cross-sectional survey method was implemented in Macao in May 2021. People aged 18 years or above and residing in Macao for 12 months prior to the study were recruited through social media. Intention to receive COVID-19 vaccines and the main constructs of the protection motivation theory and the health belief model were the main measures encompassing threat appraisal, intrapersonal characteristics, cues to action, coping appraisal, past experiences and information seeking behavior. Descriptive statistics, Pearson correlation and multiple linear regression were used for data analysis.

**Results:**

A total of 552 valid responses were received. Among the respondents, 79.5% aged between 25 and 54 years old, 59.4% were female, and 88% had a bachelor degree or above; 62.3% of the respondents indicated their intention to receive COVID-19 vaccination while 19.2% were hesitant and 18.5% did not have any intention. While 67.0% believed COVID-19 infection was life-threatening, only 19.0% thought they were at risk of getting infected. Control variables such as age, gender, education level, and having travel plans were significantly correlated with intention. Significant associations between intention with perceived severity, perceived susceptibility, maladaptive response reward, self-efficacy, response-efficacy, response cost, social attitude, social norm, past experience and information seeking behavior were identified (P < 0.05). The most important positive predictors of intention were “being able to make arrangement to receive the vaccine” (β = 0.333, P < 0.001), “a sense of social responsibility” (β = 0.326, P < 0.001), and “time off from work after vaccination” (β = 0.169, P < 0.001), whereas “concerns over vaccine safety” (β = − 0.124, P < 0.001) and “relying on online resources for vaccine information” (β = − 0.065, P < 0.05) were negative predictors. Perceived severity in terms of COVID-19 being a life threatening illness was not a predictor of intention.

**Conclusion:**

This study reaffirmed that intention to receive COVID-19 vaccination is an ongoing concern in the combat of the pandemic. Multi-component strategies to enhance health literacy that supports well-informed decision-making, increase vaccination convenience, promote social responsibility, and provide time-off incentives are among the key considerations in designing and improve vaccination campaigns in Macao.

## Background

The impact of COVID-19 pandemic on human health, economy and societal activities is unprecedented. According to the World Health Organization (WHO), since the first case was reported in the end of December 2019, there had been 188,655,968 confirmed cases of COVID-19, including 4,067,517 deaths reported as of 16th July 2021 [1]. According to the World Bank, it has been predicted that the global domestic product (GDP) would suffer a 5.2% contraction leading to a global recession and resulting in lower investments, erosion of human capital through lost work and schooling, and disruption of global trade and supply chains [2]. Health systems continued to endure massive challenges in preventing and managing the infection with COVID-19 and its variants, and be stretched to deliver effective public health measures for other infectious diseases and non-communicable diseases during critical periods [3]. On a macro level, unprecedented measures such as suspending travel, reducing crowds, and enhancing production of medical supplies had been employed across the world to mitigate the spread of COVID-19 virus. In light of the ongoing pandemic, the resilience in individual’s responsible response to COVID-19 would be just as important as “high-level” actions [4].

One of the critical public health interventions to mitigate COVID-19 pandemic at the population level is for individuals to get vaccinated with COVID-19 vaccines [5, 6]. Since the release of the genetic sequence of SARS-CoV-2 in January 2020, the research and development (R&D) activity to develop a vaccine against the disease had been intense across the globe [7]. In addition to the classic approach of inactivated viruses, the global COVID-19 vaccine R&D landscape had seen a speedy development of new-generation vaccine candidates with advanced vaccine technology paradigm such as viral vector vaccines, mRNA vaccines, and inactivated and protein subunit vaccines [8]. According to the WHO, as of 1st June 2021, there were 102 vaccines in clinical development and 185 vaccines in pre-clinical development [9]. Evidence about the safety and efficacy of COVID-19 vaccines in preventing symptomatic and asymptomatic COVID-19 infections, related hospitalizations, severe disease, or even death was mounting [10, 11]. Emerging evidence also suggested that mRNA COVID-19 vaccines might even provide protection against a variety of variant strains [12]. Moreover, marked and sustained declines in the incidence of COVID-19 infections corresponding to increasing vaccine coverage had been reported, suggesting that Covid-19 vaccination could help to control the pandemic [13]. Based on the evidence about the safety, efficacy, quality and risk management plans, drug regulatory authorities and the World Health Organization had successively listed a number of new COVID-19 vaccine candidates for emergency use [14–17].

Nevertheless, the speedy development, evaluation, approval and listing of SARD-CoV-2 vaccine do not necessarily translate into uptake of the vaccines. As of 15th July 2021, the US had administered 336.1 million COVID-19 vaccine doses and had 48.3% of the total population fully vaccinated [18]; on the same day, China had administered a total of 1414.6 million vaccine doses [19] enough to provide 707.3 million or around 48.9% of the population with 2 doses, an increase from 723.5 million doses from just 6 weeks ago on 3rd June 2021 [20]. The percentage of population fully vaccinated also varied among other developed countries (53.18% in the UK, 48.64% in Canada, 45.94% in Germany, 20.40% in Japan, 10.52% in Australia) [21]. Besides availability, accessibility and affordability of the vaccines, vaccine hesitancy is a critical challenge to national vaccination programs affecting an individual’s decision-making process about getting vaccinated [22]. Vaccine hesitancy refers to “delay in acceptance or refusal of vaccination despite availability of vaccination services”, and a high rate of hesitancy would undermine the level of demand for a vaccine [8]. The patterns of vaccine acceptance and hesitancy varied by large as reported in a systematic review that the COVID-19 vaccine acceptance rates could be high in some countries (e.g. 97% in Ecuador and 94.3% in Malaysia), but significantly lower among others (e.g. 23.6% in Kuwait and 28.4% in Jordan) [23]. Understanding the factors contributing to the individual’s intention to get COVID-19 vaccines are paramount when design effective strategies aimed to address hesitancy.

Since the onset of the COVID-19 pandemic, there had been literature investigating vaccine hesitancy and acceptance, as well as influencing factors, the source of information and its impact on acceptance, and incentives for getting vaccinated [23, 24]. At present, there is no existing scale to assess expressed intent to accept a COVID-19 vaccine nor any consensus on which theoretical framework might be ideal for this purpose. Some of this research was underpinned by different theories of health behavior such as the Oxford Covid-19 vaccine hesitancy scale (mainly assessing Covid-19 vaccine complacency, confidence and convenience) [25], the vaccine conspiracy belief scale (mainly assessing belief in conspiracy about COVID-19’s origin, covid-19 vaccines and vaccine manufacturer’s motives) [26], the health belief model (mainly assessing perceived severity, susceptibility and benefits of the vaccine, barriers to action and self-efficacy) [27], and the protection motivation theory (mainly assessing perceived severity, perceived susceptibility, maladaptive response rewards, self-efficacy, response-efficacy and response costs) [28]. Furthermore, additional measures such as cues for action, past experiences, information sources and intrapersonal characteristics such as social attitudes and social norms were also examined [29, 30]. Based on these studies, constructs outlined by these theories could be good predictors of the uptake of vaccination. However, high heterogeneity in responses about these constructs and their association with vaccine acceptance was also noted [31].

The latest estimates indicated that at least 60–75% immune individuals of a population would be necessary to halt the transmission and community spread of the virus [32, 33]. Vaccine hesitancy may impede the progress of vaccinating enough population to achieve individual protection and reach population immunity, and the decision about taking a COVID-19 vaccine is multifactorial and context-specific varying across people, time, place and vaccines [22]. There is an ongoing need to investigate the factors contributing to vaccine hesitancy or affecting the intention to get vaccinated against COVID-19. This is particularly important for areas whereby intention may be hampered by low incidence of COVID-19 cases. Therefore, the aim of this study was to investigate the intention to get vaccinated against COVID-19 and the associations between such intention and theoretically grounded and sociodemographic factors in Macao.

## Methods

An online, cross-sectional survey method was applied in this study. The project has been approved by the Panel on Research Ethics of the University of Macau in May 2021 (SSHRE21-APP018-ICMS). As clearly indicated in the Participant Information Statement in the beginning of the questionnaire, it was assumed that, by completing and submitting the survey online, they agreed to take part in the research study. Following the Strengthening the Reporting of Observational Studies in Epidemiology (STROBE) guideline [34], the reporting of the study is as follows.

### Study site and target population

Macau, with a population of around 683,100 over 32.9 km^2^, is one of the most densely populated places in the world and a famous tourist destination, exposing the city to a high risk of community transmission and imported cases amid the COVID-19 pandemic. Macau confirmed its first case of the 2019-nCoV infection on 22 January 2020 [35] and as of 16th July 2021, there were 55 confirmed cases in the city all of which were considered imported cases, with no death cases [36]. The low overall infection rate and the absence of community transmission in Macau had been largely due to the effectiveness of the border control and public health measures.

COVID-19 vaccination campaign was launched on 9 February 2021 and COVID-19 vaccines (either Inactivated vaccines or mRNA vaccines requiring two doses with 4 weeks apart) were provided free of charge to all Macao residents and non-residents who are currently studying or working in Macao. With an adequate supply of COVID-19 vaccine to cover the population ever since the launch of the vaccination campaign, an online booking system had been established for people to make appointment in advanced before visiting the designated location for vaccination according to the scheduled time. To provide additional security and guarantee for COVID-19 vaccine recipients, the government has made arrangement to provide a 1-year group insurance service to cover severe consequences caused by adverse reactions or side effects of the vaccination. On the official website of the Health Bureau, there is a page dedicated to COVID-19 vaccine information whereby daily updates about COVID-19 vaccination and adverse events following immunization are available. As of 17th July 2021, 267,742 doses of vaccines had been administer and the number of people fully vaccinated was 180,808 or nearly 30% of the population, a total of 1932 cases of adverse events (1926 minor and 6 serious cases) had been reported [37]. The target population of this survey study was people aged 18 years or above who had resided in Macao in the past 12 months. Considering the population size in Macao, a minimum sample size of 384 respondents would provide a target 5% margin of error for population percentage estimates with a level of 95% confidence.

### Design of the questionnaire

**Fig. 1:**
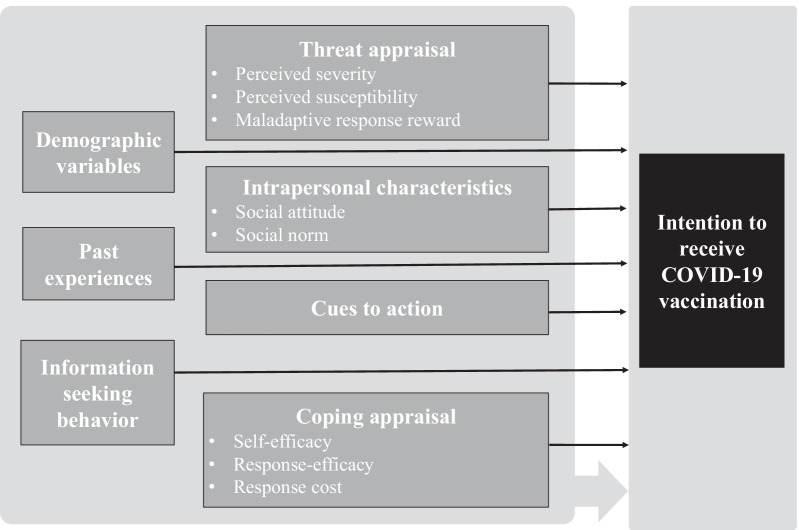
Schematic diagram of theoretically-principled constructs applied to the intention to receive COVID-19 vaccination

The design of the questionnaire was informed by (1) the current literature on intention of vaccination with COVID-19 vaccines [38–40], (2) the theoretical models commonly employed in similar studies including the health belief model [27, 41] and the protection motivation theory [29, 42, 43], and (3) a clinician involved in the local COVID-19 vaccination campaign. The schematic diagram of the theoretically informed constructs applied to the intention to receive COVID-19 vaccination I this study is provided in Fig. 1.

The questionnaire mainly comprised of two sections. Section A collected respondents’ demographic information with eight questions regarding demographic variables: age, gender, type of residency, marital status, highest level of education completed, parental status, cohabitant status, and estimated number of entries and exits to Macau in the next 6 months.

Section B asked respondents to rate their level of agreement on a set of statements using a 5-point Likert scale (1 = strongly disagree, 2 = disagree, 3 = not sure, 4 = agree, and 5 = strongly agree). There were 29 items in this section which encompassed their intention and 11 constructs: (1) perceived severity (the seriousness of the negative consequences of the health threat), (2) perceived susceptibility (the vulnerability to the negative consequences of the threatened event), (3) maladaptive response rewards (the benefits of performing the maladaptive behavior, i.e. not taking the vaccine), (4) self-efficacy (the confidence in one’s ability to successfully perform the preventative behavior), (5) response-efficacy (the effectiveness of the recommended preventative behavior in averting the occurrence of or the negative consequences of the threatened event), (6) response costs (the barriers to performance of the preventative behavior), (7) social attitude (a conditioned response to social stimuli), (8) social norms (shared expectations of acceptable behavior by groups), (9) past experiences, (10) information seeking behavior, and (11) cues for action.

At the conclusion of the questionnaire, participants were invited to provide additional feedback via a free-text response box. The questions were assigned to be mandatory answered items to avoid incompleteness and missing data.

### Development of the questionnaire

The self-administered structured questionnaire used in this study was bilingual prepared in English and Chinese in order to minimize sampling bias due to language barrier. It was validated through two pilot studies following the concepts described by Leavy [44]. The questionnaire was first pilot-tested for content validity by two doctors, two pharmacists and two researchers experienced in this research topic. All comments were taken into consideration and the questionnaire was amended accordingly. The revised questionnaires were peer-reviewed by two of the authors (COLU, HH) for comprehension and minor revisions to the wordings were made. The revised instrument was then pilot tested on a convenience sample of 10 individuals who were fluent in both languages to further assess content validity, content consistency comprehension, defective questions and the time needed to complete. Suggested changes were incorporated into the final bilingual questionnaire prior to launching on the online platform.

### Data collection

The online questionnaire, hosted by Survey Monkey, was open for 10 days from 14th May 2021 and then closed on 23rd May 2021 when no new responses were received for 24 h. Invitations to participate in the study was distributed through social media like Facebook and WeChat, and news media social platform. Given the popularity of the social media among the Macao population and the need to maintain social distancing, social media and online communication platform was chosen. The participants were recruited using a simplified-snowball sampling technique where they were invited to pass the invitation and the survey link to their contacts. The survey was estimated to take less than 8 min to complete.

### Data analysis

The data was analyzed using the Statistical Package for Social Sciences (SPSS) version 24 software for Windows [45]. In addition to descriptive statistics (frequencies, means, and standard deviations), Pearson chi-square test was used to compare the differences in COVID-19 vaccination intentions among subgroups, Spearman’s rho was used to test the correlation of intention with the variables, and multiple linear regression analysis to identity predictors of intention. Whenever the P-value is found to be smaller than 0.05, the association would be considered statistically significant at a confidence level of 95%.

## Results

### Respondents’ characteristics and the differences in the intention among subgroups

A total of 552 valid responses had been received. As shown in Table 1, the majority of the respondents aged between 25 and 54 years old (n = 439, 79.5%); more females (n = 328, 59.4%) than males (n = 224, 40.6%); more residents (n = 413, 74.8%) than non-residents (n = 123, 22.3%); more than four-fifths (n = 487, 88%) had a bachelor degree or above. In general, the participants were broadly representative of the population in Macao in terms of age and types of residency, but the proportion of female and people having higher levels of education were higher than intended.

**Table 1:** Respondents' demographic information (n = 552)

Demographic information	n	%	Intention to receive COVID-19 vaccination						X^2^	*P*
			Negative n = 102 18.5%		Not decided n = 106 19.2%		Positive n = 344 62.3%			
			n	%	n	%	n	%		
Age (years old)										
18–24	35	6.3%	9	25.7%	3	8.6%	23	65.7%	21.97	< 0.05
25–34	139	25.2%	34	24.5%	25	18.0%	80	57.6%		
35–44	157	28.4%	30	19.1%	29	18.5%	98	62.4%		
45–54	143	25.9%	23	16.1%	38	26.6%	82	57.3%		
55–64	55	10.0%	5	9.1%	6	10.9%	44	80.0%		
65+	23	4.2%	1	4.3%	5	21.7%	17	73.9%		
Gender										
Male	224	40.6%	35	15.6%	34	15.2%	155	69.2%	7.70	< 0.05
Female	328	59.4%	67	20.4%	72	22.0%	189	57.6%		
Type of residency										
Macao residents	413	74.8%	84	20.3%	88	21.3%	241	58.4%	25.88	< 0.001
Non-residents (worker)	75	13.6%	12	16.0%	13	17.3%	50	66.7%		
Non-residents (student)	48	8.7%	2	4.2%	2	4.2%	44	91.7%		
Non-residents (other)	9	1.6%	1	11.1%	3	33.3%	5	55.6%		
None of the above	7	1.3%	3	42.9%	0	0.0%	4	57.1%		
Marital status										
Married	336	60.9%	55	16.4%	70	20.8%	211	62.8%	7.32	0.293
Cohabitant	25	4.5%	7	28.0%	5	20.0%	13	52.0%		
Single	171	31.0%	37	21.6%	25	14.6%	109	63.7%		
Widowed or divorced	20	3.6%	3	15.0%	6	30.0%	11	55.0%		
Highest level of education completed										
Secondary education or below	65	11.8%	11	16.9%	22	33.8%	32	49.2%	27.67	< 0.001
Bachelor degree	267	48.4%	60	22.5%	58	21.7%	149	55.8%		
Master degree or above	220	39.9%	31	14.1%	26	11.8%	163	74.1%		
Being a parent										
No	220	39.9%	53	24.1%	36	16.4%	131	59.5%	8.22	< 0.05
Yes	332	60.1%	49	14.8%	70	21.1%	213	64.2%		
Living status										
Living alone	61	11.1%	7	11.5%	8	13.1%	46	75.4%	5.03	0.081
Living with other people	491	88.9%	95	19.3%	98	20.0%	298	60.7%		
Estimated number of entries and exits to Macau within the next 6 months										
0	196	35.5%	45	23.0%	52	26.5%	99	50.5%	24.91	< 0.05
At least once every 3 months	199	36.1%	33	16.6%	34	17.1%	132	66.3%		
At least once a month	112	20.3%	17	15.2%	18	16.1%	77	68.8%		
At least once a week	37	6.7%	7	18.9%	2	5.4%	28	75.7%		
At least once a day	8	1.4%	0	0.0%	0	0.0%	8	100.0%		

### Intention to receive COVID-19 vaccination

Overall, 334 or 62.3% of the respondents indicated their intention to receive COVID-19 vaccination, while 106 (19.2%) of the respondents were hesitant and 102 (18.5%) of respondents did not want to get vaccinated. Among respondents where were Macao residents, the percentages of positive, uncertain, and negative responses were 58.4%, 21.3% and 20.3% respectively. Among the subgroups, people aged 55–64 years old, being male, non-residents studying in Macao, being married or single, having a Master degree or above, living alone and expected to have frequent travel in and out of Macau at least once a day had higher intention to get COVID-19 vaccination. According to the Pearson Chi-square test results (Table 1), statistically significant differences in the intention were observed among the subgroups of age, gender, residency types, education level, parental status and whether having plans to travel in and out of Macao. Respondents who were 55 years old or above, male, non-residents, highly educated, being a parent or having plans to travel showed a higher intention to get COVID-19 vaccine.

### Respondents’ perception about the measurements

Descriptive statistics for items assessing factors related to intention are reported in Table 2. The results of Spearman’s rho suggested that each of the items was significantly associated with respondents’ level of intention to receive COVID-19 vaccination.

**Table 2:** Measurement of variables and other factors

		Mean	S/D	Frequency										Association with Intention to get COVID-19 vaccination	
				Strongly disagree		Disagree		Not sure		Agree		Strongly agree		Spearman's rho	*P*
				n	%	n	%	n	%	n	%	n	%		
Dependent variables															
Intention	*I intend to have COVID-19 vaccination*	3.83	± 1.41	63	11%	39	7%	106	19%	62	11%	282	51%		
PMT variables and other factors															
Severity	*COVID-19 can be a life-threatening illness*	3.82	± 1.47	78	14%	39	7%	68	12%	82	15%	285	52%	0.120	< 0.05
Susceptibility	*The risk for me to catch COVID-19 is high*	2.49	± 1.24	146	26%	146	26%	158	29%	48	9%	54	10%	0.148	< 0.001
Maladaptive response rewards (*Cronbach’s alpha 0.701*)	*If I do not get a COVID-19 vaccination, I will not have to worry about the safety of the vaccine*	2.63	± 1.35	154	28%	102	18%	168	30%	50	9%	78	14%	− 0.215	< 0.001
	*If I do not get a COVID-19 vaccination, I will not have to spend time getting vaccinated*	2.04	± 1.17	252	46%	114	21%	131	24%	24	4%	31	6%	− 0.173	< 0.001
	*If I do not get a COVID-19 vaccination, I will not have to spend money getting vaccinated*	1.72	± 1.10	337	61%	96	17%	78	14%	16	3%	25	5%	− 0.610	0.154
Self-efficacy (*Cronbach's alpha* = *0.865*)	*I am confident about my ability to make an informed decision about COVID-19 vaccination*	4.12	± 1.08	19	3%	25	5%	99	18%	133	24%	276	50%	0.468	< 0.001
	*I have the necessary information to decide whether to vaccinate against COVID-19 vaccination*	4.01	± 1.14	26	5%	32	6%	105	19%	135	24%	254	46%	0.464	< 0.001
	*I know how to register to get COVID-19 vaccination*	4.41	± 1.02	23	4%	11	2%	47	9%	109	20%	362	66%	0.442	< 0.001
	*I am able to make arrangement to get COVID-19 vaccination*	4.14	± 1.22	36	7%	31	6%	65	12%	107	19%	313	57%	0.613	< 0.001
Response efficacy (*Cronbach's alpha* = *0.921*)	*Having a COVID-19 vaccination would help reduce the symptoms if ever I contracted the disease*	3.68	± 1.22	47	9%	42	8%	116	21%	180	33%	167	30%	0.357	< 0.001
	*Having a COVID-19 vaccination would help reduce the severity of symptoms if ever I contracted the disease*	3.76	± 1.17	38	7%	38	7%	118	21%	185	34%	173	31%	0.358	< 0.001
	*Having a COVID-19 vaccination would help reduce the risk of death if ever I contracted the disease*	3.72	± 1.2	43	8%	42	8%	114	21%	182	33%	171	31%	0.371	< 0.001
Response cost	*It is likely that I will have serious side-effects that I cannot bear from COVID-19 vaccination*	3.07	± 1.22	71	13%	97	18%	187	34%	116	21%	81	15%	− 0.235	< 0.001
Social attitudes (*Cronbach's alpha* = *0.870*)	*Getting COVID-19 vaccination helps to reduce the risk of my family contracting COVID-19*	3.88	± 1.14	27	5%	35	6%	128	23%	152	28%	210	38%	0.511	< 0.001
	*Getting COVID-19 vaccination helps to prevent the diffusion of COVID-19 in the community*	4.09	± 1.06	20	4%	26	5%	90	16%	166	30%	250	45%	0.510	< 0.001
Social norm (*Cronbach's alpha* = *0.725*)	*I consider getting COVID-19 vaccination a social responsibility*	3.99	1.17	28	5%	31	6%	119	22%	114	21%	260	47%	0.655	< 0.001
	*People I know have already received COVID-19 vaccination*	4.16	1.08	18	3%	30	5%	86	16%	127	23%	291	53%	0.319	< 0.001
	*Most people I know would expect me to get COVID-19 vaccination*	3.47	± 1.28	53	10%	65	12%	164	30%	110	20%	160	29%	0.508	< 0.001
Past experience	*I have bad experiences with other types of vaccines before*	1.71	± 1.15	361	65%	72	13%	65	12%	28	5%	26	5%	− 0.150	< 0.001
Information seeking behaviour (*Cronbach's alpha* = *0.673*)	*I have actively sought information about COVID-19 vaccination*	3.48	± 1.26	50	9%	65	12%	158	29%	128	23%	151	27%	0.379	< 0.001
	*I rely on the government (such as the Health Bureau) for accurate information about COVID-19 vaccination*	3.68	± 1.27	50	9%	43	8%	132	24%	135	24%	192	35%	0.279	< 0.001
	*I rely on healthcare professionals (such as doctors, pharmacists, nurses) for accurate information about COVID-19 vaccination*	3.19	± 1.37	88	16%	81	15%	150	27%	104	19%	129	23%	0.287	< 0.001
	*I rely on online sources (such as internet, social media) for accurate information about COVID-19 vaccination*	3.68	± 1.15	34	6%	40	7%	156	28%	161	29%	161	29%	0.147	< 0.001
	*I rely on my family and friends for accurate information about COVID-19 and COVID-19 vaccination*	2.73	± 1.27	123	22%	107	19%	179	32%	82	15%	61	11%	0.085	< 0.05
Facilitating factors (*Cronbach's alpha* = *0.835*)	*I might have a stronger intention to take COVID-19 vaccination if people I know have done so*	3.42	± 1.35	74	13%	56	10%	143	26%	122	22%	157	28%	0.376	< 0.001
	*I might have a stronger intention to take COVID-19 vaccination if that somehow helps to lift travel restrictions*	3.91	± 1.3	49	9%	36	7%	92	17%	115	21%	260	47%	0.423	< 0.001
	*I might have a stronger intention to take COVID-19 vaccination if there is a rewarding system such as time off from work*	3.37	± 1.49	102	18%	54	10%	121	22%	86	16%	189	34%	0.358	< 0.001
	*I might have a stronger intention to take COVID-19 vaccination if there is financial incentive*	3.07	± 1.52	135	24%	62	11%	134	24%	68	12%	153	28%	0.265	< 0.001

*Threat appraisal*—respondents rated severity (mean = 3.82 ± 1.47) higher than susceptibility (mean = 2.49 ± 1.24). While 67% of respondents believed that COVID-19 can be a life-threatening illness, only 19% believed that the risk for them to catch COVID-19 was high. Regarding maladaptive responses, respondents rated the money they had to spend on COVID-19 vaccination the least concern (mean = 1.72 ± 1.10), followed by the time needed to get vaccinated (mean = 2.04 ± 1.17) and then worries about the vaccine safety (mean = 2.63 ± 1.35).

*Coping appraisal*—respondents rated highly their self-efficacy in terms of their ability to register for COVID-19 vaccination (mean = 4.41 ± 1.02), make appropriate arrangements (mean = 4.14 ± 1.22), their level of confidence (mean = 4.12 ± 1.08), and the information they need to make the decision about vaccination (mean = 4.01 ± 1.14). In comparison, their ratings on the response-efficacy was relatively lower regarding the effectiveness of the vaccine in reducing the severity of symptoms (mean = 3.76 ± 1.17), the risks of death (mean = 3.72 ± 1.20), and the number of symptoms (mean = 3.68 ± 1.22) in case of infection. More than one-third of the respondents believed that it would be likely for them to experience unbearable side effects after COVID-19 vaccination.

*Social attitudes and social norms*—comparatively, the respondents recognized the impact of COVID-19 vaccination on preventing the diffusion of COVID-19 in the community (mean = 4.09 ± 1.06) higher than the impact on reducing the risk of their family contracting COVID-19 (mean = 3.88 ± 1.14), with the proportion of respondents indicating positive responses differing by nearly 10%. In terms of social norms, 68% of the respondents considered consider getting COVID-19 vaccination a social responsibility and the rating was reasonably high (mean = 3.99 ± 1.17). While 76% indicated that people they knew had already received COVID-19 vaccination, only 59% of them believed that they were expected to get COVID-19 vaccination by the people they knew.

*Information seeking behavior*—only half of the respondents indicated that they had actively sought information about COVID-19 vaccination. In terms of the source of information about COVID-19 that they relied on, the government (mean = 3.68 ± 1.27) and online sources (mean = 3.68 ± 1.15) were rated the highest, followed by healthcare professional (mean = 3.19 ± 1.37) and family and friends (mean = 2.73 ± 1.27). It is worth noting that only 42% of the respondents relied on healthcare professionals for COVID-19 vaccination information, and 17% of the respondents did not rely on the information provided by the government.

*Facilitating factors*—when asked which facilitating factors might increase their intention to receive COVID19 vaccination, the respondents rated loosening of travel restrictions as a result of COVID-19 vaccination the highest (mean = 3.91 ± 1.30), followed by knowing people whom they knew having done so (mean = 3.42 ± 1.35) and a rewarding system involving time off from work (mean = 3.37 ± 1.49). The rating given to *financial incentive* was the lowest (mean = 3.07 ± 1.52)*.*

### Results of multiple linear regression

All factors found to be significantly associated with intention in Tables 1 and 2 were analyzed as the independent variables, with intention as the dependent variable. Table 3 shows the results of multiple linear regression that analyzed the relationship between intention to receive COVID-19 vaccination and the independent variables. In Model 1, the independent variables comprised only the control variables (i.e. demographic variable shown to have statistically significant correlation with intention as shown in Table 1), which only explained 11.0% of the variance in intention to receive COVID-19 vaccination. In Model 2, the independent variables comprised only the PMT factors and the control variables. A total of 45.8% of the variance in intention to receive COVID-19 vaccination can be explained by Model 2. In Model 3, the independent variable comprised all the PMT factors, other factors and the control variables. Compared to previous models, Model 3 performed best at explaining the intention to receive COVID-19 vaccination by being able to explain 57.9% of the variance in intention to receive COVID-19 vaccination.

**Table 3:** Results of multiple regression analysis

Model 1 (control variables only)					
Variables	Unstandardized coefficients		Standardized coefficients beta	t	Sig.
	B	Std. Error			
(Constant)	1.439	0.440		3.272	0.001
Control variables					
Age (years old)	0.246	0.051	0.214	4.827	0.000
Gender	− 0.244	0.117	− 0.085	− 2.081	0.038
Type of residency	0.319	0.075	0.184	4.230	0.000
Highest educational level	0.373	0.087	0.175	4.291	0.000
Being a parent	0.081	1.749	0.081	0.075	0.749
Estimated number of entries and exits to Macau in the next 6 months	0.222	0.059	0.154	**3.733**	0.000
F = 13.559, d.f. = 5, P < 0.001, R = 0.332, R2 = 0.110, adjusted R2 = 0.102					

Model 2 (PMT constructs, and control variables)						
Variables		Unstandardized coefficients		Standardized coefficients beta	t	Sig.
		B	Std. Error			
(Constant)		0.842	0.265		3.178	0.002
PMT constructs and other factors						
Perceived severity	*COVID-19 can be a life-threatening illness*	− 0.045	− 1.356	0.176	− 0.058	0.898
Perceived susceptability	*The risk for me to catch COVID-19 is high*	0.126	0.037	0.111	3.395	0.001
Maladaptive response rewards	*If I do not get a COVID-19 vaccination, I will not have to worry about the safety of the vaccine*	− 0.140	0.035	− 0.134	− 3.952	0.000
	*If I do not get a COVID-19 vaccination, I will not have to spend time getting vaccinated*	− 0.062	− 1.414	0.158	− 0.061	0.520
	*If I do not get a COVID-19 vaccination, I will not have to spend money getting vaccinated*	0.088	0.043	0.069	2.063	0.040
Self efficacy	*I am confident about my ability to make an informed decision about COVID-19 vaccination*	0.060	1.247	0.213	0.054	0.431
	*I have the necessary information to decide whether to vaccinate against COVID-19 vaccination*	0.132	0.049	0.107	2.704	0.007
	*I know how to register to get COVID-19 vaccination*	−0 .036	− 0.749	0.454	− 0.032	0.422
	*I am able to make arrangement to get COVID-19 vaccination*	0.499	0.046	0.430	10.803	0.000
Response efficacy	*Having a COVID-19 vaccination would help reduce the symptoms if ever I contracted the disease*	0.041	0.861	0.390	0.037	0.431
	*Having a COVID-19 vaccination would help reduce the severity of symptoms if ever I contracted the disease*	− 0.003	− 0.048	0.961	− 0.002	0.289
	*Having a COVID-19 vaccination would help reduce the risk of death if ever I contracted the disease*	0.182	0.041	0.155	4.417	0.000
Response cost	*It is likely that I will have serious side-effects that I cannot bear from COVID-19 vaccination*	− 0.210	0.038	− 0.182	− 5.513	0.000
*Control variables*						
Age (years old)		0.057	1.727	0.085	0.074	0.901
Gender		− 0.022	− 0.678	0.498	− 0.029	0.933
Type of residency		0.057	1.761	0.079	0.075	0.938
Highest educational level		− 0.002	− 0.045	0.964	− 0.002	0.900
Being a parent		0.045	1.394	0.164	0.060	0.942
Estimated number of entries and exits to Macau in the next 6 months		0.133	0.046	0.092	2.872	0.004
F = 57.402, d.f. = 8, P < 0.001, R = 0.677, R2 = 0.458, adjusted R2 = 0.450						

Model 3 (PMT constructs and other factors, and control variables)						
Variables		Unstandardized coefficients		Standardized coefficients beta	t	Sig
		B	Std. Error			
(Constant)		0.057	0.271		0.209	0.834
PMT constructs and other factors						
Perceived Severity	*COVID-19 can be a life-threatening illness*	− 0.033	− 1.124	0.261	− 0.048	0.911
Perceived susceptability	*The risk for me to catch COVID-19 is high*	0.086	0.033	0.076	2.607	0.009
Maladaptive response rewards	*If I do not get a COVID-19 vaccination, I will not have to worry about the safety of the vaccine*	− 0.085	0.030	− 0.082	− 2.808	0.005
	*If I do not get a COVID-19 vaccination, I will not have to spend time getting vaccinated*	− 0.012	− 0.374	0.708	− 0.016	0.768
	*If I do not get a COVID-19 vaccination, I will not have to spend money getting vaccinated*	0.041	1.386	0.166	0.059	0.880
Self efficacy	*I am confident about my ability to make an informed decision about COVID-19 vaccination*	0.064	1.819	0.069	0.078	0.631
	*I have the necessary information to decide whether to vaccinate against COVID-19 vaccination*	0.051	1.412	0.159	0.061	0.603
	*I know how to register to get COVID-19 vaccination*	0.016	0.403	0.687	0.017	0.470
	*I am able to make arrangement to get COVID-19 vaccination*	0.386	0.040	0.333	9.666	0.000
Response efficacy	*Having a COVID-19 vaccination would help reduce the symptoms if ever I contracted the disease*	− 0.018	− 0.541	0.589	− 0.023	0.672
	*Having a COVID-19 vaccination would help reduce the severity of symptoms if ever I contracted the disease*	− 0.014	− 0.429	0.668	− 0.018	0.683
	*Having a COVID-19 vaccination would help reduce the risk of death if ever I contracted the disease*	0.006	0.192	0.848	0.008	0.692
Response cost	*It is likely that I will have serious side-effects that I cannot bear from COVID-19 vaccination*	− 0.143	0.035	− 0.124	− 4.139	0.000
Social attitudes	*Getting COVID-19 vaccination helps to reduce the risk of my family contracting COVID-19*	0.051	1.384	0.167	0.059	0.564
	*Getting COVID-19 vaccination helps to prevent the diffusion of COVID-19 in the community*	0.003	0.071	0.943	0.003	0.471
Social norm	*I consider getting COVID-19 vaccination a social responsibility*	0.393	0.044	0.326	8.939	0.000
	*People I know have been already received COVID-19 vaccination*	0.003	0.094	0.925	0.004	0.648
	*Most people I know would expect me to get COVID-19 vaccination*	0.126	0.039	0.114	3.236	0.001
Past experience	*I have bad experiences with other types of vaccines before*	− 0.046	− 1.559	0.119	− 0.067	0.901
Information seeking behaviour	*I have actively sought information about COVID-19 vaccination*	0.040	1.206	0.228	0.052	0.697
	*I rely on the government (such as the Health Bureau) for accurate information about COVID-19 vaccination*	− 0.018	− 0.594	0.553	− 0.026	0.810
	*I rely on healthcare professionals (such as doctors, pharmacists, nurses) for accurate information about COVID-19 vaccination*	0.008	0.262	0.793	0.011	0.834
	*I rely on online sources (such as internet, social media) for accurate information about COVID-19 vaccination*	− 0.080	0.038	− 0.065	− 2.095	0.037
	*I rely on my family and friends for accurate information about COVID-19 and COVID-19 vaccination*	− 0.006	− 0.196	0.845	− 0.008	0.865
Facilitating factors	*I might have a stronger intention to take COVID-19 vaccination if people I know have done so*	0.025	0.691	0.490	0.030	0.591
	*I might have a stronger intention to take COVID-19 vaccination if that somehow helps to lift travel restrictions*	− 0.031	− 0.828	0.408	− 0.036	0.543
	*I might have a stronger intention to take COVID-19 vaccination if there is a rewarding system such as time off from work*	0.160	0.030	0.169	5.345	0.000
	*I might have a stronger intention to take COVID-19 vaccination if there is financial incentive*	0.060	1.255	0.210	0.054	0.337
*Control variables*						
Age (years old)		0.090	0.034	0.078	2.627	0.009
Gender		− 0.045	− 1.583	0.114	− 0.068	0.963
Type of residency		0.047	1.546	0.123	0.066	0.820
Highest educational level		0.007	0.237	0.813	0.010	0.894
Being a parent		0.016	0.500	0.617	0.021	0.753
Estimated number of entries and exits to Macau in the next 6 months		0.036	1.249	0.212	0.054	0.914
F = 82.923, d.f. = 9, P < 0.001, R = 0.761, R2 = 0.579, adjusted R2 = 0.572						

In Model 3, in addition to age, 8 items from 7 constructs were shown to be predictors of intention, including 5 positive predictors and 3 negative predictors. In the order of the strongest influence, the positive predictors were: (1) Self-efficacy—“I am able to make arrangement to get COVID-19 vaccination” (β = 0.333, P < 0.001); (2) Social norm—“I consider getting COVID-19 vaccination a social responsibility” (β = 0.326, P < 0.001); (3) Facilitating factor—“I might have a stronger intention to take COVID-19 vaccination if there is a rewarding system such as time off from work” (β = 0.169, P < 0.001); (4) Social norm—“Most people I know would expect me to get COVID-19 vaccination” (β = 0.114, P = 0.000); and (5) Perceived susceptibility—“The risk for me to catch COVID-19 is high” (β = 0.076, P < 0.05). Negative predictors were: (1) “It is likely that I will have serious side-effects that I cannot bear from COVID-19 vaccination” in response cost (β = − 0.124, P < 0.001); (2) “If I do not get a COVID-19 vaccination, I will not have to worry about the safety of the vaccine” in maladaptive response reward (β = − 0.082, P < 0.05); and (3) “I rely on online sources (such as internet, social media) for accurate information about COVID-19 vaccination” in information seeking behavior (β = − 0.065, P < 0.05).

## Discussion

This is one of the few studies that investigated the intention rate of COVID-19 vaccination in areas with low incidence rate. The intention rate in Macao was 62.3%, and age, gender, type of residency, education, parental status, and having travel plans exhibited significant correlation with intention. Importantly, it was also found that the ability to make arrangement to receive the vaccine, a sense of social responsibility, an offer of time-off from work after vaccination, peer influence, and perceived susceptibility were predictors of increased intention, while concerns about vaccine safety and seeking vaccine information from online sources were predictors of decreased intention. Together with age, these predictors could explain 57.9% of the variance in vaccination intention.

The intention rate of COVID-19 vaccination in Macao (62.3%) was higher than that reported in Hong Kong (4.2–44.2%) [46, 47], but lower than that reported in China (88.6–91.9%) [48, 49]. Higher acceptance rates of the COVID-19 vaccines had also been reported in other countries in the neighboring region (such as 94.3% in Malaysia [41], 75.8% in Australia [50] and 67.9% in Singapore [31]) and other Western countries (such as 80% in Canada [24], 75% in the US [24], 75% in Portugal [51] and 70% in Germany [51]). These intention rates indicated that hesitancy and refusal about COVID-19 vaccines is a common challenge impeding the vaccine uptake across the populations [52]. This is especially concerning for Macao considering that, since the vaccine campaign was launched in February 2021, the percentage of population fully vaccinated was only approaching 30% (as of 17th July 2021). This not only represents a significant margin from the threshold of 60–70% of the population gaining immunity to reach herd immunity, but also indicates the vulnerability of the city against the spread of the virus. Based on the factors identified in this study that were associated with vaccination intention, campaign strategies to boost vaccine uptake are further explored in the following.

### Improve health literacy to counteract the negative effect of low education background

Consistent with previous findings, older age and male gender were found to be positive factors correlated to higher rates of COVID-19 vaccine acceptance [31, 51, 53], and respondents with lower education level indicated lower vaccination intention [26, 54, 55]. Regarding the impact of education background, the literature had repeatedly demonstrated the association between lower education level and poorer health [56, 57]. The mechanism behind the association could be explained by the concept of health literary, presenting an opportunity for intervention [58]. As Biasio described, being “educated” (having some form of rigorous education) is not the same as being “literate” (being able to obtain and process information to make well-informed health decisions) [59].

Health literacy is a modifiable factor and support can be provided to improve people’s ability to engage in preventive activities and thus vaccine uptake [60, 61]. For instance, simple steps can be taken to decrease the complexity in communication and information processing. During this pandemic, in addition to detailed scientific information available on the official websites, infographic was also employed to deliver key messages to the general public [62]. Future vaccine communication strategies should consider the level of health, scientific and general literacy in subpopulations when considering the content and means of the promotion activities [31]. Continuously streamlining the COVID-19 vaccination process, and engaging healthcare professionals especially in the community level to help build public health literacy have also been recommended [61].

### *Mitigate the false sense of “*safety zone*”*

The impact of travel plans on COVID-19 vaccination intention, which was less investigated in previous studies, should be considered at least for areas which allow people mobilization among designated destinations. For instance, people in Macao and Mainland China had been allowed to travel across the border given that they had been tested negative for COVID-19 nucleic acid within 7 days prior to travel. Frequent travelers’ intention was significantly higher than the respondents who did not have any travel plans. In contrast, considering the low incidence of COVID-19 infection and the absence of community transmission, non-travelers generally felt “safe” from COVID-19 pandemic and thus perceived a low level of susceptibility affecting their intention to receive COVID-19 vaccination [63, 64]. This may also explain why, unlike previous findings [65], perceived severity was not a significant predictor of the intention to receive a COVID-19 vaccine in this study.

It is worrying because the risk of virus spread to the local community could not be ruled out. For a completely naive population, a pathogen can easily spread among susceptible hosts in a rampant manner when there is sufficient exposure of susceptible hosts to infected individuals [66]. Also, at least for the COVID-19 vaccines available to the respondents, it would take 1–2 weeks after administration of two shots of the same vaccine to reach the expected efficacy. Therefore, bringing the public’s attention to the immediate and foreseeable risks of infection and the time lag between vaccine administration and optimal protection is crucial to help them decide about taking preventative actions in a timely manner.

### Promote the social responsibility in COVID-19 vaccination and make positive action visible

In this study, 67.8% of the respondents considered getting COVID-19 vaccination a social responsibility. Our model showed that the sense of social responsibility and peer influence attributing to social norm could largely explain the variance in intention. This finding helps supplement the current literature as how social norms may influence COVID-19 vaccination has been underreported [67]. This findings also echoed to the objective in the 2012 Global Vaccine Action Plan approved by the WHO that “individuals and communities understand the value of vaccines and demand immunization as both their right and responsibility” [68].

There are at least two implications based on the findings about social norm: (1) the recognition of individual’s social responsibility should be strongly promoted within the community to leverage the power of social norm [69]; and (2) empowering vaccine recipients to share their personal stories and reasons for vaccination, and making positive decisions visible by providing vaccination badges or ribbons have been a positive influence on the decisions of others. Promoting and displaying the development momentum of social norms has been shown to be a powerful tool to shift public mentality towards health behavior change [70].

### Empower the public by optimizing their self-efficacy

Another strong positive predictor of the intention found in this study was the respondent’s ability to make arrangement needed to receive COVID-19 vaccination, reaffirming previous findings about self-efficacy being a significant positive predictor of preventive health behavioral intention [71, 72]. One important element contributed to the self-efficacy was the convenience of getting the vaccine, which may be determined in terms of the site, location and procedure. At present, to scale up from hospital site and community health clinic sites, mass vaccination sites designed to specifically address COVID-19 vaccination challenges [73] has been employed to allow high throughput. Orchestrated efforts had been made to coordinate with local communities and corporate to arrange group vaccination at the sties. Tailored vaccine-administration software systems enabled use-friendly prior booking and onsite enrolment.

Recently, group-based vaccination (e.g. corporate, employer) and out-reach vaccination sites (e.g. universities) to reach people in their communities had also been initiated. As the vaccination campaigns continue to evolve, mobile vaccination may be integrated further using mobile vaccination trucks to bring vaccination services closer to the neighboring has been to help alleviate problems with transportation, mobility, and daily schedule [74]. Importantly, the “customer journey” should be streamlined as much as possible and that requires consistent monitoring and review of the procedure. Increasing the number of vaccination sites and capacity to cater for increased vaccine demand, onsite registration and appointment rescheduling is also important [75]. There must also be a reminding system for the second dose and follow-up for no-shows. Streamlined patient experience that enabled high level of self-service and autonomy, supported by technology enablers may further maximize attendance.

### Prioritize vaccination incentives

While traditional vaccination campaign using only information and education has been increasingly seen as inadequate to reach a high vaccination rate target for COVID-19 vaccines, various forms of incentives provided by the government and business had been seen across the countries [76]. In this study, most people also agreed that they would be more willing to receive COVID-19 vaccination should there be incentives of some sort. Indeed, subsidies and other incentives are logical policy approaches to incentivize people by helping to offset the indirect costs of vaccination such as the time spent planning appointments, traveling, or waiting [77]. As effective as they are [78], incentives may also be considered as a recognition for responsible action considering the positive externalities of COVID-19 vaccination in protecting oneself as well as other people.

In terms of incentive preferences, easing travel restrictions with vaccination was considered a motivation by most people in this study, followed by an offer time off work, and financial incentives. Similarly in other countries, vaccination certificates as a way out of the restrictive travel control measures imposed by and on the city has drawn attention [79]. Suggestively, incentivizing individuals with vaccination certificates would be crucial for not only social good, but also facilitating the “recovery” of economic and socially activities as well as travel. For this, cross-jurisdiction and high-level endeavor are to engage and expand participating regions to build such infrastructure for mutual recognition of vaccination measures. More importantly, vaccine-induced protection from both infection and transmission warns more rigorous research especially with the expected emergence of variants.

Another significant incentive identified in this study was time-off from work after vaccination, which was fully reasonable at least for practical reasons. After COVID-19 vaccination, people might experience such side effects as fever, tiredness and muscle pain which might last for about 1–2 days [80]. As such, as the US-CDC recommended, it is important for employers to take strategies that appropriately evaluate and manage their employees who develop side effects after vaccination. In comparison, monetary incentive was not prioritized in this study. Indeed, the evidence about monetary incentives in changing health behavior is mixed. While a systematic review concluded that financial incentives were effective at encouraging health promoting behaviors [81], another WHO review suggested that incentive-based interventions using either conditional or non-conditional cash transfers might only cause minimal impact [82]. It is nevertheless difficult to determine the amount of money sufficient enough to drive people’s decision about vaccination at a population level, and yet such action would be highly resource consuming of which effectiveness remained questionable [83].

### Educate the public about scientific-based risk and benefit analysis about COVID-19 vaccines

Concerns about vaccine safety and information seeking behavior were found to be two negative factors in the decision making process about COVID-19 vaccination as reported previously [84]. Indeed, to encourage public acceptance for a new vaccine of COVID‐19 developed within a short period remains highly challenging [85, 86]. For the general public, misinformation regarding the vaccine safety and lack of advanced vaccination knowledge can easily contribute to anxiety, leading to overestimating possible side effects [87]. Furthermore, such low confidence in the vaccine safety could be easily inflamed by rumors and misinformation [26, 31]. In this study, while people relied on the government the most for vaccine information, it was the reliance on social media as the main source of information about COVID-19 vaccines was associated with decreased vaccine intention. As Lucia et al. concluded, concerns about the vaccine’s serious side effects complicated with misinformation contributed to the hesitancy of vaccines [88].

The need for transparency and to answer concerns about the speedy development and safety of COVID-19 vaccines based on up-to-date scientific evidence is crucial [88]. Previous experiences suggested that public messages and news releases might not be enough to convey the accurate information and to tacking false news. On that note, proactive surveillance and mitigation of the spread and harm of misinformation via social media platforms should be reinforced. Moreover, government partnering with trusted messengers (such as respected community-based groups and non-governmental organizations, formal and informal opinion leaders within these communities) to share clear, complete, and accurate messages about COVID-19 vaccines to otherwise unreachable population subgroups is also crucial [31].

The fact that less than half of the respondents found healthcare professionals their major source of vaccine information was a sign of under-utilization of healthcare resources. Resources should be targeted to empower healthcare professionals such as the pharmacists at the community settings to strengthen their capacity in having empathetic vaccine conversations, addressing myths and common questions, and providing tailored vaccine information to their patients as well as the general public [89]. Hesitant individuals may accept vaccination if reassured and provided with trustable information that the vaccine is safe and effective.

This study had a number of limitations. Firstly, using an online platform to invite participants and operating the survey online might have induced sampling bias in different ways. Through the online platform, it was not known about the population to which the invitation was distributed, making it impossible to calculate the response rate, and to fully evaluate the representativeness of the respondents. Secondly, people with lower information technology literacy might also be under-represented in this study. Thirdly, by the nature of cross-sectional study, no causal inferences could be made between the key factors and the intention based on the findings of the present study, and the factors affecting people’s intention are subject to change. Fourthly, there is a timeliness to the study findings. People’s intention to receive COVID-19 vaccination is bound to fluctuate with the spread of the virus, emergence of any evidence about vaccine safety and efficacy, new variants, etc. Lastly, the vaccination intention may be affected by factors which were not explored in this study. Until a desirable vaccination rate and herd immunity is achieved, continuous studies on people’s intention to receive COVID-19 vaccination are warranted. Follow-up studies to identify influencing factors more comprehensively, and stratified studies to determine the differences, if any, in the influences of such factors on various subgroups would be necessary for precise vaccination strategies.

## Conclusions

This study reaffirmed that low intention to receive COVID-19 vaccination is a common challenge in the combat of the pandemic. Multi-component strategies that address various factors affecting intention are needed to formulate effective interventions. For future vaccination campaign in Macao, six major strategies have been suggested based on the findings of the current study: improving health literacy of the public, helping the public come to term their vulnerability despite an apparent low incidence rate, promoting social responsibility and making positive action visible, optimizing self-efficacy of the public, reasonable prioritization of incentives, and educating the public about risk and benefit analysis of the COVID-19 vaccine. As the pandemic evolves and new evidence about the COVID-19 vaccines emerge, people vaccination intention should be continuously evaluated to drive the optimization of vaccination strategies.

## Data Availability

The datasets used and/or analyzed during the current study are available from the corresponding author on reasonable request.
